# Dosimetric Implications of Computerised Tomography-Only versus Magnetic Resonance-Fusion Contouring in Stereotactic Body Radiotherapy for Prostate Cancer

**DOI:** 10.3390/medicines5020032

**Published:** 2018-04-05

**Authors:** Daniel R. Henderson, Alison C. Tree, Kevin J. Harrington, Nicholas J. van As

**Affiliations:** 1 Royal Marsden Hospital, Fulham Road, London SW3 6JJ, UK; Alison.Tree@rmh.nhs.uk (A.C.T.); Kevin.Harrington@icr.ac.uk (K.J.H.); Nicholas.vanAs@rmh.nhs.uk (N.J.v.A.); 2 Institute of Cancer Research, 237 Fulham Road, London SW3 6JB, UK; 3 The Cancer Centre, University Hospitals Birmingham, Mindelsohn Way, Edgbaston, Birmingham B15 2GW, UK

**Keywords:** prostate, MRI, CT, fusion, radiotherapy, stereotactic, contouring, planning

## Abstract

**Background:** Magnetic resonance (MR)-fusion contouring is the standard of care in prostate stereotactic body radiotherapy (SBRT) for target volume localisation. However, the planning computerised tomography (CT) scan continues to be used for dose calculation and treatment planning and verification. Discrepancies between the planning MR and CT scans may negate the benefits of MR-fusion contouring and it adds a significant resource burden. We aimed to determine whether CT-only contouring resulted in a dosimetric detriment compared with MR-fusion contouring in prostate SBRT planning. **Methods:** We retrospectively compared target volumes and SBRT plans for 20 patients treated clinically with MR-fusion contouring (standard of care) with those produced by re-contouring using CT data only. Dose was 36.25 Gy in 5 fractions. CT-only contouring was done on two occasions blind to MR data and reviewed by a separate observer. Primary outcome was the difference in rectal volume receiving 36 Gy or above. **Results:** Absolute target volumes were similar: 63.5 cc (SD ± 27.9) versus 63.2 (SD ± 26.5), Dice coefficient 0.86 (SD ± 0.04). Mean difference in apex superior-inferior position was 1.1 (SD ± 3.5; CI: −0.4–2.6). Small dosimetric differences in favour of CT-only contours were seen, with the mean rectal V36 Gy 0.3 cc (95% CI: 0.1–0.5) lower for CT-only contouring. **Conclusions:** Prostate SBRT can be successfully planned without MR-fusion contouring. Consideration can be given to omitting MR-fusion from the prostate SBRT workflow, provided reference to diagnostic MR imaging is available. Development of MR-only work flow is a key research priority to gain access to the anatomical fidelity of MR imaging.

## 1. Introduction

Stereotactic body radiotherapy (SBRT) is a treatment option for localised prostate cancer which uses highly conformal dose distributions and precise image guidance to deliver treatment in a few large fractions [1]. A significant body of data shows that outcomes are in keeping with conventional radiotherapy and both ASTRO (American Society for Radiation Oncology) and NCCN (National Comprehensive Cancer Network) guidelines suggest SBRT as a treatment option for prostate cancer [2,3]. The profound hypofractionation used in SBRT is convenient for patients and appears to achieve similar levels of cancer control [4]. The majority of prostate SBRT centres that have published outcome data have used the Cyberknife system (Accuray, CA, USA) [4]. SBRT is currently being compared with conventional treatments in the PACE (Prostate Advances in Comparative Evidence) international phase III study [5].

MR-fusion contouring describes the process by which planning MR and CT data are fused based on prostate position. Physicians use information from both data sets to contour a target volume. However, dose calculation, image guidance and treatment processes at present use only CT data. MR-fusion contouring is considered a standard of care in prostate SBRT planning [6,7,8,9,10,11]. This is in contrast to conventional radiotherapy treatment where, typically, CT-only data are used [12,13].

The MR-fusion approach is based on studies showing that contouring with MR alone (once fusion is complete but without reference to CT) produces smaller and more consistent prostate target volumes than CT alone due to the improved soft tissue contrast, particularly at the prostate apex [14,15,16,17]. The majority of these studies included a planning component demonstrating a significant reduction in dose to the rectum and other organs, which may translate into a reduction in toxicity. This is particularly important in prostate SBRT for the 1 cc rectal constraint (typically limited to < 36 Gy, Table 1) for which higher doses are associated with increased rectal toxicity [18]. In view of the steep dose fall-off seen with SBRT, relatively small changes in prostate target volume may increase this dose significantly. Thus, there is a concern that if CT-only contours increase the prostate target volume it may make prostate SBRT impossible to plan, within current constraints.

Notwithstanding the above, there are a number of objections to the use of MR-fusion contouring in prostate SBRT planning. First, the fusion process itself is subject to variability in accuracy of between 1–4 mm [19,20,21] (setting aside the inherent difficulty in measuring fusion accuracy). This may negate the benefits as, for example, reported differences in apex position between MR-only and CT contouring are in this range [15,22]. Furthermore, differences in bowel and bladder filling between the two data sets may alter prostate and seminal vesicle shape and position [23]. Second, more recent studies of MR-only volumes have shown that these may be relatively similar to those produced with CT, due to the increased use of diagnostic MR and the resultant increase in physician awareness of CT over-estimation [22,24]. Finally, the need to perform an additional planning MR scan significantly increases resource use and adds to workflow. As MR-fusion is not typically used in conventional radiotherapy, setting up a prostate SBRT service or clinical trial may be limited by this, reducing patient access.

As the majority of patients with localised prostate cancer can expect good disease control with acceptable toxicity, the probability of demonstrating incremental clinical benefit using MR-fusion compared with CT-only contours is low. For this reason, there is very unlikely to be a clinical study examining this comparison. However, whether CT-only volumes are significantly different enough to have a dosimetric impact in SBRT is unknown. We investigated this question, in order to determine whether there is a strong enough justification to continue to mandate MR-fusion contouring in prostate SBRT.

## 2. Materials and Methods

We retrospectively compared prostate target volumes and SBRT plans for patients treated clinically with MR-fusion contouring (standard of care) with those produced by re-contouring using CT data only. Our institution is an experienced SBRT centre treating patients with localised prostate cancer since 2011. Our hypothesis was that CT-only volumes would be larger, such that planning within accepted SBRT rectal and bladder constraints would not be possible for all patients.

**Figure 1:**
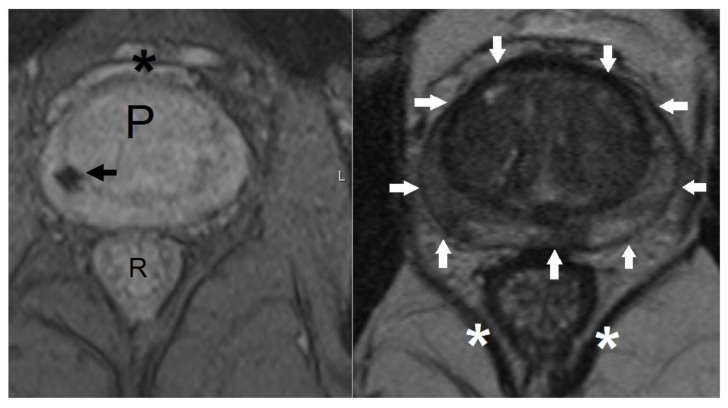
T2-weighted magnetic resonance (MR) sequences used for fusion with computerised tomography (CT) planning scan. Axial gradient echo (left pane) and T2 fast spin echo (right pane) MR images at the level of the prostate. Left pane: low signal void (black arrow) represents the site of a fiducial marker. P, prostate; R, rectum; *, venous plexus of Santorini. Right pane: white arrows show position of prostatic capsule. *, levator ani muscles.

**Figure 2:**
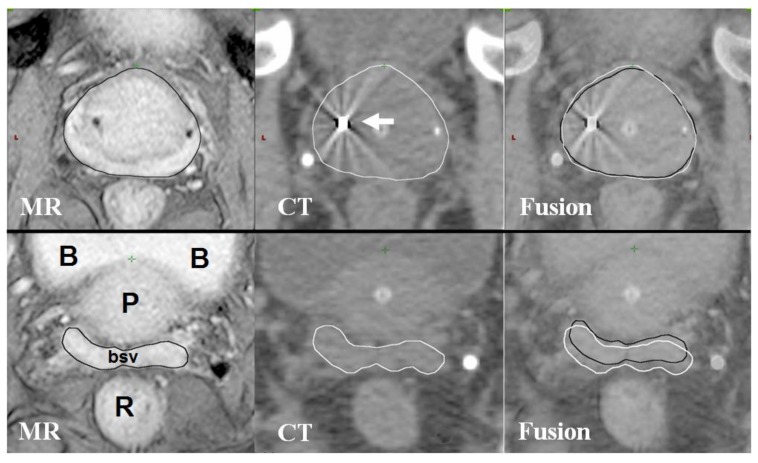
MR and CT fusion for prostate contouring.Axial MR and CT images at the level of the prostate (upper three images) and base of seminal vesicles (lower three images). B, bladder; P, prostate; bsv, base of seminal vesicles; R, rectum. White arrow shows gold fiducial marker on CT image. MRI-based contours are in black, CT-based contours are in white. The upper three images demonstrate good fusion of prostate MRI and CT imaging; lower three images from the same patient showing less accurate fusion at the base of seminal vesicles. The difference in position of bsv can be seen in the fusion images where contours from MRI (black) and CT (white) are shown overlapping.

Planning CT data sets from twenty consecutive patients previously treated with SBRT for localised prostate cancer were used. Ethical approval for data collection and processing was given as part of a Service Evaluation by our Institutional Service Evaluation Committee. Ethical approval code: SE24, date of approval: 1 August 2013. Patients recruited to this study gave written informed consent prior to enrolment. Treatment planning had been done as per PACE phase III trial protocol (NCT01584258). Patients initially had four 1 × 3 mm cylindrical gold fiducial markers inserted into the prostate under transrectal ultrasound guidance. One week later, patients had planning CT and MR scans on the same day. Micro-enemas were given for two consecutive days before and 1–2 h prior to the CT scan. Patients were asked to drink approximately 200 mL of water 1 h prior to the CT scan, in order to achieve a “comfortably full” bladder. Scans were taken using the Lightspeed RT16 system (General Electric, Boston, MA, USA) with a 1 mm slice thickness. MR scans were taken following the CT scan using the Magnetom Aera 1.5 T system (Siemens, GmbH, Munich, Germany) with 3 mm slices. Two T2-weighted images were taken, one fast spin echo sequence to define the prostate capsule and one gradient echo sequence to identify fiducial marker position (Figure 1). Fusion of the MRI and CT planning scans was done based on fiducial marker position using the Eclipse (Varian Medical Systems, Palo Alto, CA, USA) radiotherapy planning system. The prostate and base of seminal vesicles (bsv) were contoured using the fused images, to form the clinical target volume (CTV). The base of seminal vesicles was defined as the proximal 1 cm of seminal vesicles, measured from their attachment to the prostate. During MR-fusion contouring, the image is windowed between MR and CT, therefore, data from both image sets are used. This is useful as there may be differences in position and shape of the prostate, which may occur due to fusion accuracy or differences in bowel and bladder filling between the two scans. For example, fusion can be less accurate at bsv, as this site is further from the fiducial markers than the prostate itself (Figure 2). Organs-at-risk (rectum, bladder, bowel, femoral heads and penile bulb) were contoured using the planning CT only. Contours were imported into the Multiplan inverse planning software for the Cyberknife SBRT system version 5.1.2 (Accuray Inc., Sunnyvale, CA, USA). Planning criteria are specified in Table 1 [4]. These criteria have been used by the majority of Cyberknife centres [8,11,25,26]. As such, the PTV (planning target volume) was formed by adding a 5 mm circumferential margin with 3 mm posteriorly. The prescription dose was 36.25 Gy in five fractions, typically prescribed to the 80% isodose.

For this study, using the CT data set only, the CTV was re-contoured. Reference to the original (MR-fusion) contours or diagnostic MR scan was not permitted. The CT data sets were presented anonymously for contouring to a physician experienced with prostate and SBRT contouring. This process was repeated two months later, in order to assess intra-observer variability. Contours were also reviewed by another experienced physician to reduce inter-observer variability. The final volume on each occasion was therefore a consensus between the two observers. The original MR-fusion contours were labelled “MRF,” the two CT-only contours (done two months apart) “CT1” and “CT2.” CTV volumes were calculated for MRF, CT1, CT2 and the position of the contoured prostate apex was recorded. The Dice similarity coefficient was used to compare the spatial concordance of volumes. Dice coefficient = 2 * (V1 ∩ V2)/(V1 + V2), that is, the intersection (∩) of the volumes to be compared (V1 and V2) multiplied by two, divided by the sum of those volumes. A value of 0 indicates a complete absence of overlap, a value of 1 indicates that the volumes are identical. Patients were then re-planned by a trained Cyberknife planner who had not previously been involved with the cases using contours from CT1, with no reference to the original plans. A successful plan was considered one which met all dose constraints with minor variations only (Table 1). The primary outcome of interest was the rectal V36 Gy constraint, which is typically the most challenging to meet. This specifies that the volume receiving 36 Gy or above should be less than 1 cc (or 2 cc with a minor variation). In our experience, the majority of patients planned have a V36 Gy close to 1 cc. Therefore, a relatively small increase in V36 may mean it is not possible to achieve a successful plan. Sample size was limited by practicalities however: 20 patients give 81% power to detect a 0.5 cc mean difference in rectal V36 Gy with a significance of 0.05 (two tailed paired *t*-test; standard deviation 0.75 cc). In our judgement, a difference below 0.5 cc would not produce clinically significant differences in plans. The paired *t*-test was used for comparisons. Statistics were calculated using SPSS version 20 (IBM, Armonk, NY, USA). Supplementary File S1 contains the raw data for the study.

## 3. Results

**Figure 3:**
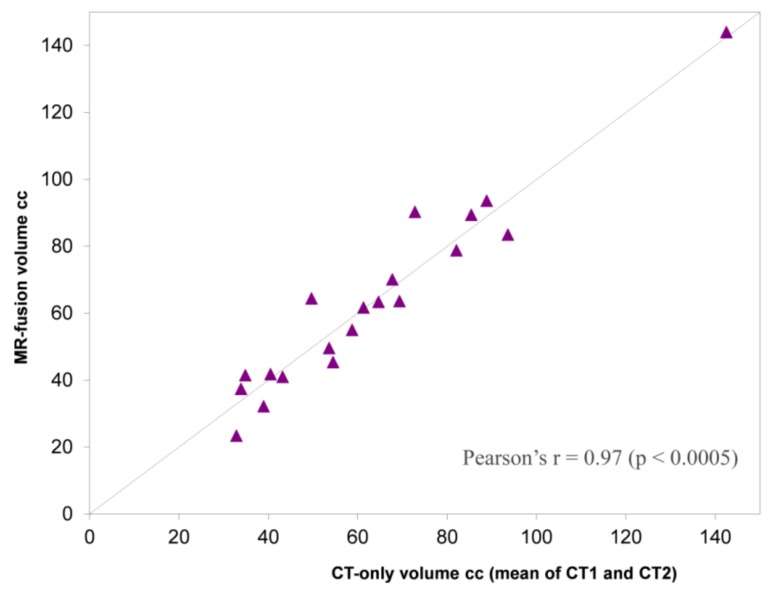
Plot of mean CT-only volume (CT1 and CT2) against MR-fusion volume. Dashed line: volumes equivalent. To left of dashed line MR > CT volume. To right of dashed line CT > MR volume.

There were no significant differences between MR-fusion contoured and CT-only contoured CTVs (Table 2 and Figure 3). The mean Dice coefficients for MR-fusion contoured CTVs compared with CT-only CTVs were 0.86 (±0.04) and 0.85 (±0.05) for CT1 and CT2, respectively (Table 3). Comparing the two CT-only volumes (to determine contouring consistency), the Dice coefficient was 0.92 (±0.02). On average the prostate apex was contoured 1.1 mm (±3.5; −0.4–2.6) more inferiorly on the MR-fusion contours compared with CT-only. The prostate base was contoured, on average, 1.2 mm (±2.7; 0.0–2.3) more inferiorly (Table 4).

In nineteen of 20 patients, it was possible to achieve a successful plan using both MR-fusion and CT-only contours. In one patient, it was not possible to achieve a PTV V36.25 Gy above 90% due to prostate volume, with either MR-fusion or CT-only contours. This was due to a very large prostate (144 cc). However, the PTV V36.25 Gy identical (83%) for both MR-fusion and CT-only contours. The mean PTV V36.25 Gy was 96% (±3.0) for MR-fusion contoured plans and 96% (±3.0) for CT-only plans. Table 5 shows the comparison between doses to organ-at-risks in the MR-fusion contoured and CT-only plans. There were no significant differences in rectal V18.1 Gy and bladder V37 Gy. Small statistically significant differences in favour of CT-only plans were seen in the rectal V36 Gy and also the rectal V29 Gy and bladder V18.1 Gy.

## 4. Discussion

Our study showed no dosimetric detriment of using CT-only contouring compared with MR-fusion contouring. As can been seen from Table 5, there were some statistically significant differences in certain constraints. However, the magnitude of these differences was very small and therefore not clinically significant. In particular, the rectal V36 Gy constraint, for which our study was powered to detect a 0.5 cc difference, was slightly lower in the CT-only group. PTV coverage was identical for MR-fusion contours and CT-only contours implying that PTV coverage was not being compromised to ensure adequate rectal V36 Gy. To the best of our knowledge, this is the only study to examine how CT-only contouring compares with MR-fusion contouring in prostate SBRT planning.

Our findings stand in contrast to three studies investigating this question in conventionally fractionated radiotherapy with fused planning CT and MR imaging. Debois et al. contoured prostate CTVs using CT alone, followed by MR alone one week apart. In 10 patients, they found that MR-only volumes were smaller and that the resultant plans showed a 20% reduction in rectal V80% [15]. Steenbakkers et al. compared CT-only and MR-only volumes in 18 patients. MR-only volumes were smaller and associated with an approximately 3–5 Gy lower equivalent uniform dose to the rectal wall [16]. Finally, Sannazzari et al. showed similar findings in an 8 patient study [17]. These studies in conventional radiotherapy generally used larger PTV margins than SBRT (around 10 mm).

There are a number of possible reasons why MR-fusion and CT-only contoured volumes were similar in this study, in contrast to these previous studies. First, it is important to note that, once the fusion process was complete, these studies compared contours derived from MR data alone to those from CT alone. MR-fusion contours use data from both and allow appreciation of fusion discrepancies and changes in prostate shape and position due to bowel and bladder filling. In view of the fact that CT is used for dose calculation and treatment planning, contours are likely to be expanded to account for these differences, meaning MR-fusion volumes will be larger than MR-only volumes. Second, two studies have shown similar volumes with MR-only and CT-only contouring. Both Usmani et al. (40 patients) and Parker et al. (8 patients) found no significant difference in absolute volumes [22,24]. These studies did not include a planning component. The authors suggested that increasing physician awareness of MR prostate anatomy and how this relates to CT anatomy, may be responsible for this finding. This is consistent with the increasing use of diagnostic MR for prostate cancer.

Our dosimetric findings are perhaps unsurprising, given that the absolute CTV volumes and Dice coefficients were very similar. The finding of a Dice coefficient of 0.92 (±0.02) for CT1 and CT2 CTV contours shows that intra-observer variability was low. CT1 and CT2 volumes were reviewed by a separate observer to reduce inter-observer variability.

We do acknowledge, that this is a single-institution study and would be strengthened by independent validation. However, our findings should be widely applicable as the majority of centres have access to diagnostic MR imaging to consult while contouring. Furthermore, although our sample size was larger than the previous planning studies discussed, 20 patients is a relatively modest number and larger studies would more precisely define any differences.

Our results suggest that MR-fusion is not necessary to successfully plan prostate SBRT and therefore consideration can be given to omitting this. Thus, an additional planning MR and the time taken for fusion could be avoided. Furthermore, as investigators in the PACE phase III study (NCT01584258), which aims to recruit more than 800 patients to the radiotherapy arms, we have amended the protocol to no longer mandate MR-fusion.

It is important that this change is justified in terms of volume accuracy (and potential geographical miss). Table 3 shows that the Dice coefficient demonstrated that MR-fusion and CT-only volumes were not spatially identical. Table 4 shows, in keeping with previous studies, that variability occurred at the base and apex. Assuming MR-fusion contouring represents the gold standard, one objection to the CT-only approach would be that a portion of the prostate might be missed, resulting in clinical detriment. It is noted that, at prostatectomy, positive margins typically occur at the apex [27]. Against this, the Cyberknife system reports a sub-millimetre accuracy for treatment delivery [28]. Thus, the typical PTV margin of 5 mm (3 mm posteriorly) is larger than that required to compensate for treatment accuracy errors alone and may therefore negate small differences in CTV volume. Furthermore, turning to clinical data, Loblaw et al. have reported on 84 patients treated on a standard linear accelerator with prostate SBRT using CT only for contouring [29]. A 4 mm PTV margin was used. At 55 months median follow-up, this group reported excellent toxicity and cancer-control outcomes. Finally, although using larger margins, large trials in conventional radiotherapy where CT-only contours are used have shown good long-term disease and toxicity outcomes [12,13]. However, it is important to state that PTV margins should be present to account for setup variability (and potential organ motion), rather than suboptimal contouring.

What do our results mean for MR imaging in prostate SBRT radiotherapy planning? First, it is clear from multiple studies that MR-only contours reduce inter-observer variability and are likely to result in more accurate contours [15,16,22,24]. Second, unlike CT, MR imaging can identify dominant disease foci within the prostate itself and allow dose-escalation to this area. This focal dose-escalation approach has the potential to improve outcomes and is being investigated in clinical trials [30]. However, our findings suggest that to gain access to these benefits, there is a need for MR-only workflow. Contouring, planning and delivery would then be based on a single image set with excellent soft tissue contrast. The technology for this is currently being developed in the MR-linac [31] (Elekta, Stockholm, Sweden) and MRIdian [32] (Viewray, Oakwood, OH, USA) systems. However, at present, these technologies require a CT to determine electron density data. A key research priority is to develop a reliable method to determine electron density from MR data, in order to remove the need for CT. The ultimate aim is to use the imaging modality with the highest anatomical fidelity as best practice for contouring in prostate radiotherapy.

## 5. Conclusions

In this study, CT-only contours were similar to MR-fusion contours with no dosimetric detriment, suggesting that single-modality workflow is appropriate. Consideration can be given to omitting MR-fusion from the prostate SBRT workflow, provided reference to diagnostic MR imaging is available. This study also highlights the opportunity for MR-only workflow, which is currently being developed for MR-linac systems.

## Figures and Tables

**Table 1:** Prostate SBRT dose constraints and planning objectives (derived from PACE phase III trial).

Parameter	Constrain/Target	Minor Variations
PTV	V36.25 Gy ≥ 95%	90–94.9%
CTV (prostate + bsv)	V40 Gy ≥ 95%	90–94.9%
CTV-PTV margins	5 mm, with 3 mm posteriorly	-
Rectum	V18.1 Gy < 50% V29 Gy < 20% V36 Gy < 1 cc	- - ≥1 cc but ≤2 cc
Bladder	V18.1 Gy < 40% V37 Gy < 10 cc	- ≥10 cc but ≤20 cc

bsv, base of seminal vesicles; SBRT, stereotactic body radiotherapy; PACE, Prostate Advances in Comparative Evidence (NCT01584258); CTV, clinical target volume; PTV, planning target volume.

**Table 2:** CTV volumes.

Volume	Mean Volume cc (±SD)	*p* Value vs. MRF
MRF	63.5 (±27.9)	-
CT1	63.2 (±26.5)	0.84
CT2	63.8 (±26.7)	0.89

CTV, clinical target volume; MRF, MR-fusion; CT1 and CT2, CT-only volumes drawn two months apart.

**Table 3:** Dice coefficients.

Volumes Compared	Mean Dice Coefficient (±SD)
MRF vs. CT1	0.86 (±0.04)
MRF vs. CT2	0.85 (±0.05)
CT1 vs. CT2	0.92 (±0.02)

MRF, MR-fusion CTV; CT1 and CT2, CT-only CTVs drawn two months apart.

**Table 4:** Difference in superior-inferior prostate apex and base positions for MR-fusion compared with CT-only contours.

Position	MRF vs. CT1	MRF vs. CT2	CT1 vs. CT2
Mean difference in apex position (mm ±SD; 95% CI)	1.1 (±3.5; −0.4–2.6)	1.1 (±3.1; −0.3–2.4)	−0.1 (±2.1; −1.0–0.9)
Mean difference in base position (mm; ±SD; 95% CI)	1.2 (±2.7; 0.0–2.3)	1.7 (±3.5; 0.1–3.2)	0.3 (±1.8; −0.5–1.1)

Negative numbers indicate CT-only contours are more inferior with respect to MRF contours. MRF, MR-fusion contours; CT1 and CT2 CT-only contours drawn two months apart.

**Table 5:** Organ-at-risk doses for MR-fusion and CT-only plans.

		MR-Fusion	CT-Only	Comparison	
Organ	Constraint *	Mean Volume Receiving ≥ Constraint (±SD)		Mean Difference (95% CI)	*p* Value
Rectum	V18.1 Gy (<50%)	33% (±9.2)	28% (±8.9)	5.0% (−0.1–10)	0.05
	V29 Gy (<20%)	11% (±3.2)	9.4% (±2.5)	1.7% (0.3–3.1)	0.02
	V36 Gy (<1–2 cc)	1.3 cc (±0.5)	1.0 cc (±0.4)	0.3 cc (0.1–0.5)	0.02
Bladder	V18.1 Gy (<40%)	26% (±9.3)	21% (±8.5)	4.8% (1.6–8.3)	0.01
	V37 Gy (<10 cc)	6.2 cc (±2.6)	5.3 cc (±2.2)	0.9 cc (−0.1–1.88)	0.08

* Constraints from PACE phase III prostate SBRT trial (see Table 1).

## Supplementary Materials

The following are available online at http://www.mdpi.com/2305-6320/5/2/32/s1, File S1: Supplementary appendix with source data.

## References

[1] Benedict S.H., Yenice K.M., Followill D., Galvin J.M., Hinson W., Kavanagh B., Keall P., Lovelock M., Meeks S., Papiez L. (2010). Stereotactic body radiation therapy: The report of aapm task group 101. Med. Phys..

[2] NCCN National Comprehensive Cancer Network—Clinical Practice Guidelines in Oncology: Prostate Cancer. http://www.nccn.org/professionals/physician_gls/pdf/prostate.pdf.

[3] ASTRO Model Policies: Stereotactic Body Radiotherapy. https://www.astro.org/uploadedFiles/Main_Site/Practice_Management/Reimbursement/2013HPcoding%20guidelines_SBRT_Final.pdf.

[4] Henderson D.R., Tree A.C., van As N.J. (2015). Stereotactic body radiotherapy for prostate cancer. Clin. Oncol..

[5] ClinicalTrials.gov (2017). Prostate Advance in Comparative Evidence (Pace)—nct01584258.

[6] Madsen B.L., Hsi R.A., Pham H.T., Fowler J.F., Esagui L., Corman J. (2007). Stereotactic hypofractionated accurate radiotherapy of the prostate (sharp), 33.5 gy in five fractions for localized disease: First clinical trial results. Int. J. Radiat. Oncol. Biol. Phys..

[7] Boike T.P., Lotan Y., Cho L.C., Brindle J., DeRose P., Xie X.J., Yan J., Foster R., Pistenmaa D., Perkins A. (2011). Phase i dose-escalation study of stereotactic body radiation therapy for low- and intermediate-risk prostate cancer. J. Clin. Oncol..

[8] Chen L.N., Suy S., Uhm S., Oermann E.K., Ju A.W., Chen V., Hanscom H.N., Laing S., Kim J.S., Lei S. (2013). Stereotactic body radiation therapy (sbrt) for clinically localized prostate cancer: The georgetown university experience. Radiat. Oncol..

[9] Bolzicco G., Favretto M.S., Satariano N., Scremin E., Tambone C., Tasca A. (2013). A single-center study of 100 consecutive patients with localized prostate cancer treated with stereotactic body radiotherapy. BMC Urol..

[10] Katz A.J., Santoro M., Diblasio F., Ashley R. (2013). Stereotactic body radiotherapy for localized prostate cancer: Disease control and quality of life at 6 years. Radiat. Oncol..

[11] Oliai C., Lanciano R., Sprandio B., Yang J., Lamond J., Arrigo S., Good M., Mooreville M., Garber B., Brady L.W. (2013). Stereotactic body radiation therapy for the primary treatment of localized prostate cancer. J. Radiat. Oncol..

[12] Lee W.R., Dignam J.J., Amin M.B., Bruner D.W., Low D., Swanson G.P., Shah A.B., D’Souza D.P., Michalski J.M., Dayes I.S. (2016). Randomized phase iii noninferiority study comparing two radiotherapy fractionation schedules in patients with low-risk prostate cancer. J. Clin. Oncol..

[13] Dearnaley D., Syndikus I., Mossop H., Khoo V., Birtle A., Bloomfield D., Graham J., Kirkbride P., Logue J., Malik Z. (2016). Conventional versus hypofractionated high-dose intensity-modulated radiotherapy for prostate cancer: 5-year outcomes of the randomised, non-inferiority, phase 3 chhip trial. Lancet Oncol..

[14] Hentschel B., Oehler W., Strauss D., Ulrich A., Malich A. (2011). Definition of the ctv prostate in ct and mri by using ct-mri image fusion in imrt planning for prostate cancer. Strahlenther. Onkol..

[15] Debois M., Oyen R., Maes F., Verswijvel G., Gatti G., Bosmans H., Feron M., Bellon E., Kutcher G., Van Poppel H. (1999). The contribution of magnetic resonance imaging to the three-dimensional treatment planning of localized prostate cancer. Int. J. Radiat. Oncol. Biol. Phys..

[16] Steenbakkers R.J., Deurloo K.E., Nowak P.J., Lebesque J.V., van Herk M., Rasch C.R. (2003). Reduction of dose delivered to the rectum and bulb of the penis using mri delineation for radiotherapy of the prostate. Int. J. Radiat. Oncol. Biol. Phys..

[17] Sannazzari G.L., Ragona R., Ruo Redda M.G., Giglioli F.R., Isolato G., Guarneri A. (2002). Ct-mri image fusion for delineation of volumes in three-dimensional conformal radiation therapy in the treatment of localized prostate cancer. Br. J. Radiol..

[18] Elias E., Helou J., Zhang L., Cheung P., Deabreu A., D’Alimonte L., Sethukavalan P., Mamedov A., Cardoso M., Loblaw A. (2014). Dosimetric and patient correlates of quality of life after prostate stereotactic ablative radiotherapy. Radiother. Oncol..

[19] Amdur R.J., Gladstone D., Leopold K.A., Harris R.D. (1999). Prostate seed implant quality assessment using mr and ct image fusion. Int. J. Radiat. Oncol. Biol. Phys..

[20] Kagawa K., Lee W.R., Schultheiss T.E., Hunt M.A., Shaer A.H., Hanks G.E. (1997). Initial clinical assessment of ct-mri image fusion software in localization of the prostate for 3D conformal radiation therapy. Int. J. Radiat. Oncol. Biol. Phys..

[21] Seppala T., Visapaa H., Collan J., Kapanen M., Beule A., Kouri M., Tenhunen M., Saarilahti K. (2015). Converting from ct- to mri-only-based target definition in radiotherapy of localized prostate cancer: A comparison between two modalities. Strahlenther. Onkol..

[22] Parker C.C., Damyanovich A., Haycocks T., Haider M., Bayley A., Catton C.N. (2003). Magnetic resonance imaging in the radiation treatment planning of localized prostate cancer using intra-prostatic fiducial markers for computed tomography co-registration. Radiother. Oncol..

[23] Kerkhof E.M., van der Put R.W., Raaymakers B.W., van der Heide U.A., van Vulpen M., Lagendijk J.J. (2008). Variation in target and rectum dose due to prostate deformation: An assessment by repeated mr imaging and treatment planning. Phys. Med. Biol..

[24] Usmani N., Sloboda R., Kamal W., Ghosh S., Pervez N., Pedersen J., Yee D., Danielson B., Murtha A., Amanie J. (2011). Can images obtained with high field strength magnetic resonance imaging reduce contouring variability of the prostate?. Int. J. Radiat. Oncol. Biol. Phys..

[25] Tree A.C., Ostler P., Hoskin P., Dankulchai P., Nariyangadu P., Hughes R.J., Wells E., Taylor H., Khoo V.S., van As N.J. (2014). Prostate stereotactic body radiotherapy—First uk experience. Clin. Oncol..

[26] King C.R., Brooks J.D., Gill H., Presti J.C. (2011). Long-term outcomes from a prospective trial of stereotactic body radiotherapy for low-risk prostate cancer. Int. J. Radiat. Oncol. Biol. Phys..

[27] Smith J.A., Chan R.C., Chang S.S., Herrell S.D., Clark P.E., Baumgartner R., Cookson M.S. (2007). A comparison of the incidence and location of positive surgical margins in robotic assisted laparoscopic radical prostatectomy and open retropubic radical prostatectomy. J. Urol..

[28] Kilby W., Dooley J.R., Kuduvalli G., Sayeh S., Maurer C.R. (2010). The cyberknife robotic radiosurgery system in 2010. Technol. Cancer Res. Treat..

[29] Loblaw A., Cheung P., D’Alimonte L., Deabreu A., Mamedov A., Zhang L., Tang C., Quon H., Jain S., Pang G. (2013). Prostate stereotactic ablative body radiotherapy using a standard linear accelerator: Toxicity, biochemical and pathological outcomes. Radiother. Oncol..

[30] Tree A., Jones C., Sohaib A., Khoo V., van As N. (2013). Prostate stereotactic body radiotherapy with simultaneous integrated boost: Which is the best planning method?. Radiat. Oncol..

[31] Raaymakers B.W., Lagendijk J.J., Overweg J., Kok J.G., Raaijmakers A.J., Kerkhof E.M., van der Put R.W., Meijsing I., Crijns S.P., Benedosso F. (2009). Integrating a 1.5 t mri scanner with a 6 mv accelerator: Proof of concept. Phys. Med. Biol..

[32] Mutic S., Dempsey J.F. (2014). The viewray system: Magnetic resonance-guided and controlled radiotherapy. Semin. Radiat. Oncol..

